# A new gas detection technique through cross-correlation with a complex aperiodic FBG

**DOI:** 10.1038/s41598-024-59841-7

**Published:** 2024-04-30

**Authors:** Matthew Rahme, Peter Tuthill, Christopher Betters, Maryanne Large, Sergio Leon-Saval

**Affiliations:** 1 Sydney Astrophotonics Instrumentation Laboratory, School of Physics, The University of Sydney, Sydney, NSW 2006 Australia; 2 Institute of Photonics and Optical Science, School of Physics, The University of Sydney, Sydney, NSW 2006 Australia

**Keywords:** Fiber Bragg gratings, Cross-correlation spectroscopy, Optical filters, Gas detection, Nonlinear optics, Optical sensors, Infrared spectroscopy, Fibre optics and optical communications, Integrated optics

## Abstract

Optical cross-correlation is a technique that can achieve both high specificity and high sensitivity when deployed as the basis for a sensing technology. Offering significant gains in cost, size and complexity, it can also deliver significantly higher signal-to-noise ratios than traditional approaches such as absorption methodologies. In this paper, we present an optical cross-correlation technology constructed around a bespoke customised Fiber Bragg Grating (FBG). Exploiting the remarkable flexibility in design enabled by multiple aperiodic Bragg gratings, optical filters are devised that exactly mimic the absorption features of a target gas species (for this paper, acetylene $C_2H_2$) over some waveband of interest. This grating forms the heart of the sensor architecture described here that employs modulated optical cross-correlation for gas detection. An experimental demonstration of this approach is presented, and shown to be capable of differentiating between different concentrations of the $C_2H_2$ target gas. Furthermore these measurements are shown to be robust against interloper species, with minimal impact on the detection signal-to-noise arising from the introduction of contaminant gases. This represents is a significant step toward the use of customised FBGs as low-cost, compact, and highly customisable photonic devices for deployment in gas detection.

## Introduction

The science of spectroscopy owes much of its central place in optical physics to its ability to reveal details of chemical composition and physical environment from the analysis of signals encoded with wavelength upon a beam of light. However apparatus to perform the measurements can be cumbersome and expensive; producing a relatively large volume of data (spectra) that must be distilled to what are often only a select number of desired quantities (eg. concentrations). Techniques based on optical cross correlation offer avenues to circumvent some of these difficulties. Rather than sensing by matching unknown sample data to library spectra by computer, this step is instead accomplished optically prior to the photosensor. This can greatly reduce requirements on the volume of data collected while allowing for a simpler, more efficient device that can perform with no loss in sensitivity. The key enabling technology for the novel sensor explored in this paper is an optical Fibre Bragg Grating (FBG).

FBGs have found widespread application across science and industry since their invention nearly a half century ago. Their optical response is highly sensitive to both strain and temperature, making them ideal for use to sense changes in environment in many contexts. In addition to their high sensitivity, they are also largely immune to high-voltage electrical fields, favouring their deployment in conditions where traditional electronic sensors would fail^1^. From material strain monitoring to sonar, FBGs have already been demonstrated to be extremely versatile and cost-effective solutions^2–5^. Of recent interest is their growing use in chemical monitoring, where they offer a low-cost, compact, yet highly sensitive options in the development of new gas detectors^2,5,6^.

Despite strong demand arising from myriad applications across science and industry, widespread adoption of miniaturised gas sensors has been hampered by the inability to meet the twin demands of sensitivity and specificity. High sensitivity using direct absorption (DA) methods demands large, expensive and complex spectrometers; smaller devices generally require compromise and trade-offs that limit sensitivity^7^. To increase the selectivity of a detector, an ideal approach is to monitor multiple absorption features simultaneously. By targeting many features, sensitivity can be increased while simultaneously the effects of non-target gas species can be minimised, with specificity dramatically enhanced by matching a more complete “fingerprint” unique to the desired chemical. Our method to create an optical template with which to perform this match employs a FBG fabricated to encode multiple aperiodic Bragg wavelengths^8^. This FBG has been customised so that its Bragg wavelengths provide an excellent match the absorption bands of the target gas. Put another way, our custom FBG acts as a “photonic molecule” engraved in glass fibre: an optical simulacrum capable of exactly mimicking the spectral features of the target gas species over the wavelength range of interest.

A system employing a notch filter has been modelled by Cheriton et al.^9^, who demonstrated recovery of the IR spectrum of Venus. Various groups have independently pursued architectures to deliver improved sensors based on the ability of FBGs to create complex wavelength-selective filters, including the work of Tuthill^10^ and Sivanadam et al.^11^, however prior to the present paper, a major challenge has been in the fabrication of the required bespoke tailored FBG to exactly match spectral response of the target molecule.

However the writing and demonstration of customised FBGs with the desired characteristics is not without precedent. Indeed such devices, fabricated so as to reproduce (in absorption) atmospheric molecular emission lines, have shown promising results and the potential to offer new capabilities in the field of Astrophotonics^12^. This use of complex aperiodic FBGs, configured as an optical filter in the near-infrared region of the spectrum, have been deployed to reject specific molecular emission lines. The sum total of many (of order a hundred) bright but narrow lines occurring at quasi-regular intervals combine to contaminate a spectral window important to ground-based astronomy. This near-infrared bright sky background problem^13^ is predominantly caused by the relaxation of hydroxyl (*OH*) molecule’s rotational and vibration modes in the upper atmosphere. Widely regarded as on of the intractable problems plaguing ground-based infrared astronomy for decades, this bright and highly variable telluric emission has presented significant barrier in studying faint astronomical objects with important signatures in the near-infrared—from galaxies to exoplanets.

Spectrographs that incorporate bespoke FBGs that knock out the problematic OH lines are now being developed; specifically the GNOSIS and later PRAXIS instruments both deploy customised FBGs capable of suppression of more than 100 OH emission lines simultaneously. Configured as optical notch filters, they reject unwanted background radiation in reflection, transmitting only clean interline regions of the spectrum through to the science detector. GNOSIS^14^ demonstrated that the targeted *OH* lines could be suppressed by 30 dB, improving the interline background by a factor of 4. PRAXIS^15^ was further able to improve the SNR in the interline regions between the targeted lines by up to a factor of 17. Although these devices did not involve the direct detection of any gases, they pioneered fabrication of FBGs that could be written to replicate any given spectral fingerprint. Building upon this foundation, these same design and fabrication technologies for creation of custom FBG devices are here carried over into a new application: an active sensor of novel architecture purposed for gas sensing and analysis.

Aperiodic FBGs with transmission notches customised to match molecular absorption features make an ideal matched-filter for use in an optical gas detection sensor based on cross-correlation methods^16^. Optical Cross-correlation Spectroscopy (OCS) achieves significantly higher SNRs by framing the measurement as a differential signal. This can be accomplished by modulating the response of the spectral matched-filter (conceptually, the location of the wavelength notches in the filter transmission profile). For spectroscopic measurement such techniques are used to increase measurement sensitivity over limits obtained by traditional DA methodologies by calibrating for and removing sources of background noise and variation^11^. The use of analogous differential techniques have been demonstrated to great effect in a number of astronomical measurement settings. However, traditional spectroscopic data is taken using large, highly complex and costly instruments, such as echelle spectrographs^11^. Simpler solutions to this problem were explored decades ago^17,18^; however, again these devices were still too complex and costly to develop into general-purpose gas detectors.

Whilst OCS is a promising technique for gas detection the key challenge regarding its implementation is the realisation of the spectral filter. Many optical techniques and configurations to generate the complex featured wavelength response have been trialled. Varying levels of success have been demonstrated by approaches ranging from multi-wavelength gratings, Lyot filters^19^, Fabry-Perot cavities^9,20^ and Michelson interferometry^21^. Of particular note are filters generated from silicon waveguides^22^ and ring resonators^23,24^ which produce periodic filters with the ability to shift wavelength features. A key feature of almost all these approaches is that, to keep the optical setup relatively simple, the wavelength response of the filter produced typically results in regularly spaced notches at a repeating cadence dictated by the physics of the optical apparatus. Whilst these types of filters can be effective over limited ranges for chemicals with pseudo-periodic absorption features, they lack the flexibility of customised FBGs. The match they provide to the true molecular response is limited both in precision and in wavelength extent. What is really needed is the capability for producing photonic molecules that exactly match complex spectra over wide spectral ranges: a capability that FBGs stand ready to deliver.

Another advantage of employing complex FBGs for OCS is their modest footprint makes it straightforward to operate several or many in parallel, scaling up the possibilities so that multiple FBGs can be incorporated into a single device. For example an architecture which also incorporates photonic lanterns—fibre devices that enable the coupling of multimode fibres to multiple single mode fibres^25^—would be one way to upscale the capabilities of a sensor. Based on these technologies, devices could embody several or many complex FBGs implemented into “multi-line” gas detectors^26^ all operating in parallel so that the eventual specificity and complexity of possible filter combinations is almost unlimited. In addition, the use of lanterns enables the strictly single-moded technology of complex FBGs to be used in a setting where light collection by way of multimode fibres is desired, for example for reasons of better coupling for light collection.

Here we present the first complex FBG written to mimic the absorption profile of a target gas using a novel writing technique^10^; specifically, the P-band absorption lines of acetylene ($C_2H_2$) have been used for the purposes of this demonstration. Furthermore, an apparatus constructed around this grating that delivers gas detection using cross-correlation techniques is described. A successful demonstration of the apparatus and the methods delivered data capable of determining the relative concentration of gases contained in various standard cells. Furthermore we explore the impact of contaminant gases, comparing our experimental results with simulation. As shown in the following sections, these results together constitute a significant step toward an architecture for the deployment of FBGs as low-cost, compact photonic devices for gas detection with high sensitivity and specificity.

### Cross-correlation spectroscopy: theory

Cross-correlation spectroscopy is usually performed by translating a template filter across spectral features of interest. For simplicity, we might envisage the filter to consist of multiple notches, each tailored to target corresponding molecular features. As the filter is scanned through wavelength, its varying overlap with spectral features from the molecular structure means that the transmitted intensity will be modulated. Using the model presented by Sivanandam et al.^11^ the transmitted intensity for a given spectral feature can be expressed as:

1 $$\begin{aligned} C(v) = S(\lambda )T(\lambda ) \end{aligned}$$

Where *T* is the transmission profile of the notch filter, *S* is the spectrum of the target object, *C* is the observed cross-correlation lag. Here $v = \Delta \lambda /\lambda _0*c$ where $\Delta \lambda$ is the offset from the rest wavelength and $\lambda _0$ is the wavelength that corresponds to the spectral feature in question. Overall, the transmitted intensity will be maximised when the notch filter is most closely aligned with the series of corresponding molecular spectral features that it was designed to mimic. When using an FBG, we have the advantage of having a well defined transmitted and reflected signal. Therefore, at the same location one obtains maximum transmission we will witness a minimum in the reflected signal: that is when the filter and the spectral features are aligned. Hence, the sensor should ideally be constructed so as to monitor both transmitted and reflected ports of the FBG, where an intensity maximum and minimum, respectively would then be expected as the filter passes through alignment with the molecular spectrum.

To achieve this optical cross-correlation—i.e. simulated modulation spectroscopy—all structure in the template filter must be modulated simultaneously. Two possible methods to accomplish this suggest themselves: varying the temperature or the tension of a complex FBG can produce the required modulation. This is because the Bragg wavelength ($\lambda _B$) of an FBG is affected by both temperature and applied strain by an amount ($\Delta \lambda _B$) that can be expressed as^27^:

2 $$\begin{aligned} \frac{\Delta \lambda _B}{\lambda _B} = (\alpha _s + \alpha _e)\Delta T + (1-p_e)\epsilon \end{aligned}$$

where $\alpha _s$ is the thermal expansion coefficient, $\alpha _e$ the thermo-optic coefficient, $\Delta T$ the temperature change, $p_e$ the effective strain and $\epsilon$ the strain. If we adopt strain as our primary method of tuning the grating (so that the temperature is kept stable – $\Delta T = 0$) then Eq. (2) simplifies to:

3 $$\begin{aligned} \Delta \lambda _B = (1-p_e)\epsilon \lambda _B \end{aligned}$$

This equation quantifies the relationship between varying the strain applied to the FBG and the resulting modulated response in the Bragg wavelengths. Because the change occurs simultaneously for all Bragg wavelengths, this mechanism can be used to perform cross-correlation gas measurements over all of the features written into our complex FBG.

## Methods

### Novel complex FBG structure

Gratings in fibres are made by imprinting a section of varying refractive index that is specifically tailored to the desired Bragg wavelength(s). The technology for writing gratings into fibres is mature and involves generating an interference pattern in UV light. Sufficiently intense UV light incident on glass generates a slight change in refractive index. Various methods to produce the desired interference pattern are well established and can involve: interferometric, phase mask, and point-by-point methods^28^. Whilst these techniques have been used to write single-wavelength FBGs for decades, aperiodic gratings with multiple Bragg wavelengths are significantly more complex. Here we utilise a design process that treats all N channels simultaneously (as employed for the GNOSIS spectrograph; a detailed description is contained in Bland-Hawthorn et al.^12^).

**Figure 1:**
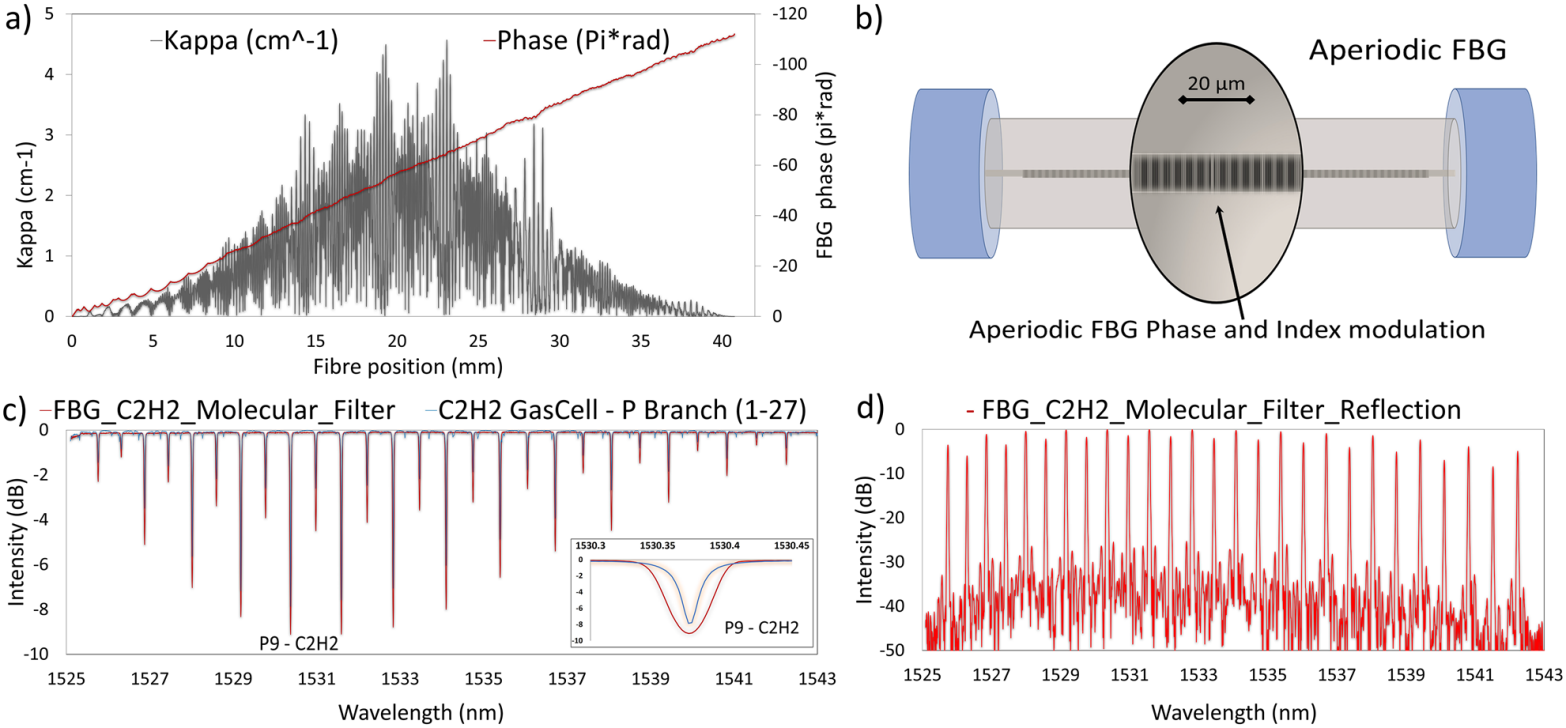
(**a**) Custom design of the aperiodic FBG as represented by amplitude ($\kappa$) and phase ($\theta$) along the axis of the fibre. (**b**) Schematic representation of the index modulation and phase variation in the core of a complex aperiodic FBG optical fiber. (**c**) Comparison of the complex FBG transmission response (red) with the p-band absorption features of $C_2H_2$ (black) which it was designed to mimic. (**d**) Reflection spectrum of the complex FBG filter.

To fabricate our aperiodic multi-notch FBG with complex kappa amplitude and phase design, a versatile interferometry-based inscription setup using a Sagnac interferometer and acousto-optic modulators (AOM) was used. The aperiodic complex FBG period and index modulation intensity (Kappa) design can be seen in  Fig. 1a. In our system, the laser used is the Innova FreD (Frequency-Doubled) ion laser from Coherent, generating 244 nm continuous-wave laser light. The phase mask generates a diffraction pattern, and its $\pm \,\,1\textrm{st}$ order forms a Sagnac loop. The phase of each beam inside the loop is modulated by two AOMs individually. The resultant index modulation can be tuned by both period and magnitude independently (as shown schematically in  Fig. 1b). This enables the capability of central wavelength detuning which permits the inscription of complex structure into the fiber core^29^. The resultant transmission and reflection spectra can be seen in  Fig. 1c and d, respectively. The grating was written to mimic the P branch absorption features of $C_2H_2$ and the strong overlap can be seen in the insert of  Fig. 1b, c with all major features aligned. This constitutes (to the best of our knowledge) the first such device reported in the open literature.

### Optical testbed and data analysis

A fibre-coupled benchtop Superluminescent Diode (SLD; ThorLabs model S5FC1005P with centre $\lambda$ 1550 nm, bandwidth 50 nm, power 22 mW, T: 22.5 $^\circ$C) was used as the light source for the experiment. The SLD was coupled to a calibrated reference gas cell or to multiple cells in series. The light was then passed through the complex FBG, which was placed on a large copper block connected to a heater and kept at a constant temperature of ($24.5 \pm 0.1 ^\circ C$) for all experiments. A photodetector was used to monitor transmitted light while an optical circulator enabled a second detector to simultaneously monitor the reflected signal. The detector outputs were recorded using the data logger function on a Moku-Pro precision oscilloscope (50,000 samples/s).

**Figure 2:**
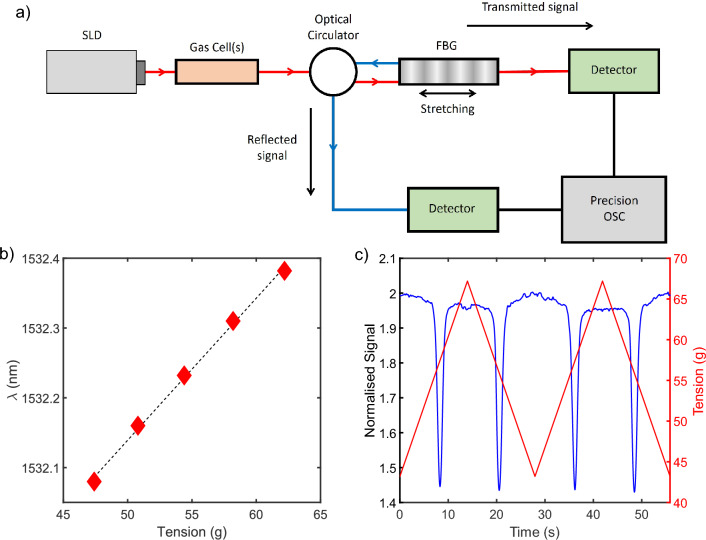
(**a**) Schematic of the experimental setup. Transmitted (red) and reflected (blue) light paths are shown. The fibre is stretched using clamps and a motor to precisely control the strain. (**b**) The wavelength of a single peak from the complex FBG spectrum as a product of applied strain. The observed shift exhibits a direct proportionality to the applied strain, underscoring a consistent correlation between the strain and the wavelength alteration. (**c**) Example trace using a single $C_2H_2$ gas cell showing the intensity of reflected light (blue) as the tension applied (red) to the FBG is modulated. This shows the total intensity of reflected light as stretching occurs.

The aperiodic FBG was secured in place, clamped at each end. One clamp was attached to a linear translation stage moving under computer control with a 25 mm actuator. The applied strain was measured using a scale (calibrated in grams) attached to the stage. This setup can be seen in Fig. 2a). Motor retractions increased tension on the complex FBG structure, resulting in the grating wavelength response shifting longwards, as expected from Eq. (3). Figure 2b shows the shift of a single peak to longer wavelengths as the tension is increased. A change in tension will shift all of the Bragg wavelengths. The exact shift will vary slightly for each peak, being dependant on the design of the device, temperature variability, and how evenly the strain is applied. However, variation between peaks is negligible compared to the overall shift, as will be highlighted later.

Following from this, modulating the motor position allowed all the Bragg wavelengths to periodically cycle in and out of alignment with the absorption peaks of the P band of $C_2H_2$. Examining the total reflected intensity then enabled this effect to be carefully monitored. As seen in Fig. 2c, when tuned so that the peaks coincide, the intensity of reflected light decreases, as expected.

To characterise the optical response of different $C_2H_2$ concentrations, standard calibrated gas reference cells were employed. Three fibre coupled cells of increasing $^{12}C_2H_2$ concentration were used: NIST SRM2517a (6.7 kPa, 5.5 cm path length), the output reference from the ANDO AQ6317B spectrum analyser, and NIST SRM2517a (6.7 kPa, 3 cm path length). For ease of reference the NIST SRM2517a will be labelled as the tested reference, since simulations were performed using the spectrum from this cell. The other two cells will be referred to as reduced cells 1 and 2 respectively. Gas cells of $^{12}CO$ (Wavelength References CO-12-200, 26.7 kPa), and $H^{13}C^{14}N$ (NIST SRM2519, 13.3 kPa, 5.5 cm path length) were also employed to test the effect of contaminant gases. When testing multiple gases simultaneously cells were concatenated in series allowing the accumulated spectra to emulate mixed chemical environments.

The positions of each peak were tracked with custom software (written in MATLAB), wherein a Gaussian profile was fitted to each to obtain the intensity. To account for different coupling losses between each cell, the signal was normalised to a baseline level defined to occur between each second peak. Peaks were analysed two at a time to account for the background asymmetry that arises from the hysteresis observed with motor direction. This asymmetry is noticeable in Fig. 2c.

As noted above, both the transmitted signal and the reflected signal can be used to obtain the final science output: the gas concentration. The reflected signal, however, had a higher signal-to-noise ratio, so it was used for the measurements in this study. To obtain a measure of concentration using this reflected signal, the difference between the baseline and the fitted location of each minima was used: a greater magnitude indicated a higher gas concentration.

### Simulating the effect of contaminant gases

The impact of unwanted gases in a mixed environment together with the target species was explored by comparing the reflection spectra of the FBG to $C_2H_2$ in the presence of *CO* and *HCN*. Designating these latter two as “interlopers”, for this section the combined optical effects of all gases was projected from measured data on each individually using MATLAB code. Both absorption and FBG spectra were converted from dB to a linear scale to match the measured voltage scale used above and kept as simple vector arrays. Only a segment of the full absorption spectra obtained for $C_2H_2$ was used (1525.5–1540 nm). A corresponding segment was also taken from the FBG spectrum with an offset applied to the index arrays. The offset was increased, while the wavelength vector was not changed, to simulate a strain-induced shift in the Bragg grating response.

For a given offset the molecular absorption array was multiplied by its corresponding value in the FBG template array, and this new array was summed. This summed value can then be used as a metric for the total light reflected by the FBG array. The results were then normalised using the same method as described above for the measured data. This procedure was then repeated after adding the absorption spectra of “interlopers” *HCN* or *CO* to that of $C_2H_2$. A weighting factor was also applied to account for coupling losses in the experimental segment of this study. The weighting factor was determined by measuring the direct output loss of each component.

## Results

### Differing concentrations

**Figure 3:**
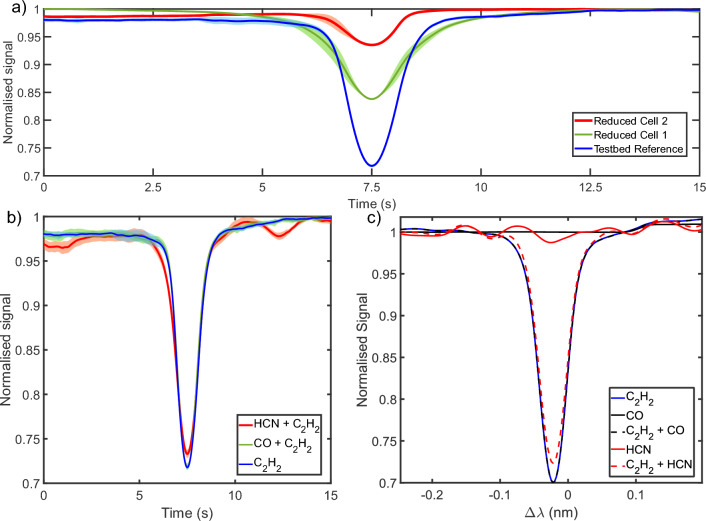
(**a**) Averaged and normalised sweeps for three different gas cell concentrations of $C_2H_2$. Increasing concentration correlates with lower minima for each gas chamber. (**b**) OCS signals for $C_2H_2$ compared to mixed gas configurations that also include *CO* and *HCN* as ‘contaminants’. The shaded regions in both graphs represent the standard deviation ($\sigma$) of the averaged sweeps. (**c**) Simulation results for $C_2H_2$ and contaminating gases, including those in mixed gas environments. The simulations involving *HCN* and $C_2H_2$ exhibit features in the interline period that closely mirror those observed in the experimental data presented in the previous panel.

In this section, we outline the use of the FBG to sense different concentrations using standard gas cells and to test the impact of contaminant gases. The presence of the target gas $C_2H_2$ was probed by modulating the applied tension to the custom FBG. Recall from the above that when the Bragg wavelengths align with the p-band $C_2H_2$ absorption, a minimum in reflected intensity occurs. Establishing the relative concentration of each gas cell can be done by measuring the magnitude of each minima after normalisation. As the concentration of the $C_2H_2$ was increased a positive trend was observed in the magnitude of the measured dip. This clear trend is seen in Fig. 3a), with the most concentrated cells producing a stronger drop in the reflected signal when the customised FBG and molecular absorption features are in tune.

We also noticed variation in the signal recorded, most notably in the baseline. To account for this, we recorded 43 sweeps over a 10 minute window, repeating for each gas cell configuration and combination. In the first two panels of Fig. 3 we show the average (solid lines) and standard deviation (shaded region) for each configuration. In an initial comparison of sweeps, an asymmetry was observed between alternating sweeps, attributable to differences in motor direction. Consequently, to calculate the average and standard deviation, only one motor direction was used. However, it is pertinent to mention that this variation is unlikely to stem from motor control. The 25 mm actuator has a resolution of 40 nm and a minimum step size of 50 nm. The minimum step in the wavelength of the grating can be deduced by taking into account two factors: firstly, the tension shift is 24 g/mm, and secondly, based on regression analysis performed on the data presented in Fig. 2b, the wavelength shift amounts to 20.4 nm/pg. Given the linear nature of all responses, a minimum step size of 153 fm is derived. As this value significantly falls below the detection limit of the utilised equipment, it is unlikely to contribute to any observed variation.

Overall while there was notable variability in the level of the baseline signal (regions where the filter is detuned), there was little deviation in the magnitude of each minima ($<3\%$ for all measured configurations). Furthermore, this stability indicates that any variation in the shift of individual Bragg wavelengths is minimal when compared with the overall shift of the grating, as expected. Since the magnitude of the minima is the primary key to determination of gas concentration, the low variability in the measured data is encouraging for the eventual translation of this technology to a calibrated sensor.

**Figure 4:**
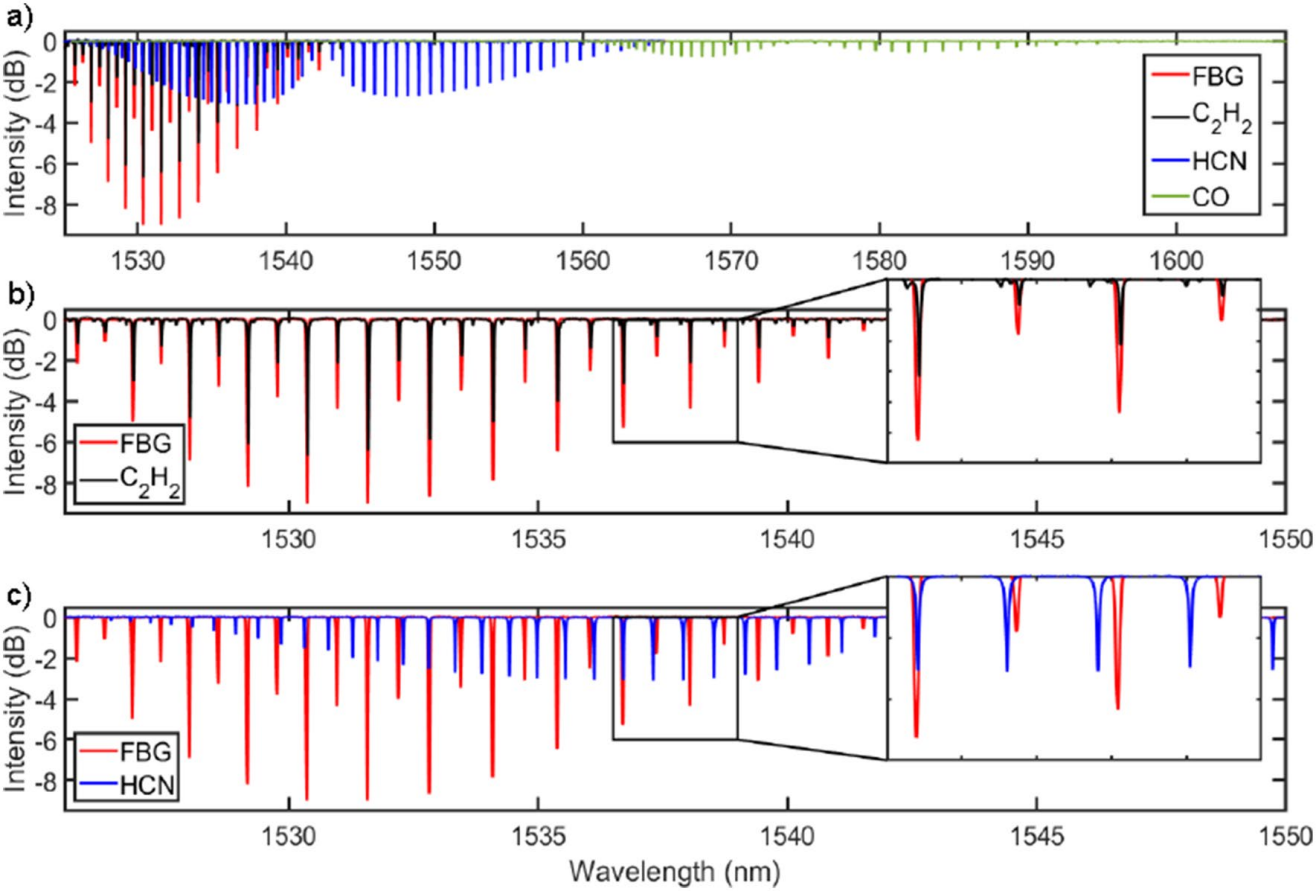
(**a**) Transmission spectrum of the complex FBG (red) compared with the absorption spectra of $C_2H_2$ (black), *HCN* (blue), *CO* (green). (**b**) Absorption spectrum of $C_2H_2$ (black), compared with the transmission of the complex FBG (red). (**c**) Absorption spectrum of *HCN* (blue), compared with the transmission of the complex FBG (red).

### Impact of other gases

$C_2H_2$ was chosen for this study as it is an ideal test molecule with strong pseudo-periodic absorption features over a wavelength range convenient for our optical fibre technologies. It is also a common standard for optical calibration, so that a range of calibrated chambers are readily available for testing.

Establishing a clear trend between concentration and intensity of the reflected light from the FBG constitutes a significant step toward establishing the basis of such devices in gas detection. However, chemical monitoring is almost always performed in impure, mixed gas environments. We tested the capabilities of our custom FBG template to operate in such conditions using CO and HCN as ‘contaminant’ gases. As before, multiple sweeps of the OCS apparatus were averaged and combined to produce our data plots. These tests were first performed individually with single gas cells in isolation, and later cells were grouped in series together with the highest concentration $C_2H_2$ cell.

Turning first to signals arising from the CO alone, no clear peaks are observed over the range scanned. This indicates that we should expect minimal impact from *CO* as an interloper species. Indeed, we see from Fig. 4a that there is no overlap in absorption features between $C_2H_2$ and those of *CO* in the region of interest, making it an ideal control gas. The sweeps performed with only $C_2H_2$ returned a minima at $0.732 \pm 0.003$, which was within error of the sweeps with both *CO* and $C_2H_2$ which returned a minima of $0.741 \pm 0.010$. This measurement also provided a validation of the simulation, which indicated a difference of less than $0.1 \%$ between a pure and mixed environment, which is well within the error of the measured results. In summary, we anticipated minimal impact from *CO*, and our results did not show detectable influence.

Next, we turn to *HCN* which presents a far more challenging case for our sensor as this contaminant gas is expected to have some impact on the measured concentrations of the target. The R and P absorption branches of *HCN* overlap with the P branches of $C_2H_2$, and as a consequence, they also overlap with our template FBG. This can be clearly seen in Fig. 4c: some features overlap almost directly while a significant number lie between those of $C_2H_2$. It is also important to note that the linewidth of individual peaks created by the FBG template is larger than that of the target gases. Therefore, the potential for confusion and unwanted signals generated by this interloper species is somewhat under-represented by simple comparison of the spectra at their native molecular linewidths, as in Fig. 4. For all these reasons, disentangling *HCN* from target $C_2H_2$ presents an excellent challenge in recovering the measured signals from our candidate sensor.

OCS signals were first recovered from a setup incorporating only a *HCN* standard cell. As in the earlier case with *CO*, no discrete narrow peaks were present over the scanned range, although unlike the previous case, here a prominent variation was observed. The simulation of the correlation spectrum arising from pure *HCN* shows the variation given by the red trace in Fig. 3c. Experimental signals obtained from ganged cells simulating a mixed gas environment still preserved the strongly peaked signal when strain brings the sensor in tune with the $C_2H_2$ target. However, it can also be seen from the figure that variations from *HCN* persist in the background, and indeed their shape is largely preserved except near the peak where $C_2H_2$ dominates. A difference of $3.7 \pm 1.0 \%$ was measured between a pure $C_2H_2$ and a mixed *HCN* and $C_2H_2$ configuration. The variation in the background is consistent across all scans, and the close match with the simulated traces. As expected, the outcome is then that the basic robust detection made by the sensor suffers little impact, although influence from the interloper is clearly present. This is consistent with the simulated response, which predicted that the presence of *HCN* would produce a difference of order $3.3 \%$: an excellent match to the measured difference in normalised intensity. We further note that these results imply that should the presence of the contaminant be known in advance, or determined from the shape of the signals, then the effects are entirely predictable and therefore could in principle be calibrated out, ameliorating any impact on sensing the target.

Although they do not alter the underlying conclusions, there are a few caveats to be aware of with regard to these simulation and experimental outcomes. First, the spectra used in the simulation are from data obtained from a setup whose resolution is lower than the intrinsic spectral linewidth of each peak. Second, the simulation does not account for noise arising from instability in the imposed strain (arising from imperfections in the motorised actuator). In addition, these tests do not account for inhomogeneous spectral broadening, that would occur in a mixed environment, owing to testing being performed in series. However, despite these minor points, the close match between expectations and experimental results is an encouraging step in validating sensor performance in multi-species environments. The fact that the observed signals were overwhelmingly dominated by resonance with the target gas bodes well for accurate measurements of concentration, despite the presence of contaminants. Furthermore, the finding that the background variation for the blended *HCN* experiment could be well simulated indicates that more information, and perhaps even the sensing of gases not directly tailored into the grating, could be recovered from the OCS data in future work.

## Conclusions

We have presented results from the first optical testbed to perform optical cross-correlation spectroscopy using an aperiodic fibre Bragg grating. This complex FBG was custom designed and shown to yield an excellent match to a $\sim \,\,20$ nm region of the p-band absorption spectrum of $C_2H_2$. As an optical simulacrum of the molecule itself, it provides the ideal matched-filter template to perform optical cross-correlation, probing for the presence of the target species. Acting as a complex notch filter and employing strain as a mechanism to cyclically tune/detune the device from resonance with the molecular spectrum, we demonstrated that our device is capable of distinguishing varying levels of concentrations of the target gas. Our prototype sensor also exhibited robust insensitivity to the presence of unwanted contaminant gases. Overall, our findings indicate the viability of customised FBGs as compact, cost-effective devices for use in the spectral fingerprinting of specific target gases.

In future work, we aim to apply this technique to other gases of interest targeting, for example, the pressing need for methane sensors for use in environmental monitoring. Whilst the present work focused on relative measurements and detection/non-detection scenarios, next generation experiments should enable the calibrated determination of the optical path integrated molecular count. The compact, lightweight and robust nature of the photonic componentry employed for this testbed augurs well for the future potential integration into portable detectors for real-world sensing applications.

## Data Availability

Data underlying the results presented in this paper are not publicly available at this time, but may be obtained from the first author upon reasonable request.
